# Social context and moderate-severe food and nutritional insecurity in families with children aged 0-59 months, Paraíba, Brazil, 2017-2018

**DOI:** 10.17843/rpmesp.2023.401.12328

**Published:** 2023-03-29

**Authors:** Dixis Figueroa-Pedraza

**Affiliations:** 1 Universidade Estadual da Paraíba, Campina Grande, Paraíba, Brasil. Universidade Estadual da Paraíba Universidade Estadual da Paraíba Campina Grande Paraíba Brazil

**Keywords:** Food Security, Child, Social Vulnerability, Social Support, Social Environment, Brazil

## Abstract

**Objective.:**

To analyze the association between the social context (demographic, socioeconomic and social support factors) and moderate-severe food and nutritional insecurity in families with children aged 0-59 months enrolled in municipal kindergartens in the state of Paraíba, Brazil.

**Materials and methods.:**

We conducted a cross-sectional study in Brazilian municipalities prioritized for the prevention of childhood obesity. A questionnaire was used to collect information on the social context of the family (demographic profile of the child, socioeconomic situation and social support) as well as the Brazilian food insecurity scale. The association between the independent variables and moderate-severe food and nutrition insecurity was determined by applying Poisson regression to estimate crude and adjusted prevalence ratios and their respective 95% confidence intervals.

**Results.:**

We included 382 families; 27.2% had moderate-severe food and nutrition insecurity. In addition, dysfunctional families with children under 24 months, from less affluent classes, beneficiaries of the Bolsa Família Program and without social support (material, emotional/informational and interaction) were more likely to present the outcome.

**Conclusions.:**

Our results show that 27.2% of the families had moderate-severe food and nutritional insecurity, were beneficiaries of the Bolsa Família Program, dysfunctional and did not have social support. Therefore, the identification of these factors would be useful to improve family food and nutritional security.

## INTRODUCTION

Ensuring food and nutrition security (FNS) is a critical global goal, both for the sustainable development of nations and for the promotion of the nutritional well-being and health of populations ^1^. However, its implementation at the local level has been insufficient, since there were still 811 million people in the world facing famine in 2020, most of them children. Estimates from the same year suggest a worrying nutritional scenario for children under five years of age: 149 million stunted, 45 million underweight and almost 39 million overweight ^2^. These figures show that a great effort needs to be made in order to achieve the global eradication of hunger and the different forms of malnutrition, including Brazil as a signatory of such proposals ^1^^,^^2^. In 2018, one fifth of Brazilian families were in a situation of hunger ^3^. Furthermore, in 2019, 7.0%, 3.0% and 13.1% of children under five years of age were undersized, thin and overweight, respectively ^4^.

Food and nutrition insecurity (FNI) is associated with decreased food intake, inadequate child feeding practices and poor health conditions ^5^. In Brazil, FNS implies that the population should have guaranteed access to food, with adequate quantity and quality, without compromising other essential needs ^6^. In fact, there are several factors that determine the levels of FNI, with low buying power and lack of access to nutritious food being the most predominant factors ^7^^-^^9^.

A systematic review with meta-analysis of articles published between 2004 and 2013 reported that the prevalence of FNI among Brazilian populations with social inequities was 87.2%, evidencing social determination ^10^. Likewise, for samples obtained from schools and kindergartens, the same study reported that 61.8% of families were in the same situation ^10^, despite the role of the National School Feeding Program in ensuring FNS of students in the public education system ^11^. Thus, Brazilian studies on FNS have identified socioeconomic factors as important determinants of this problem ^6^^,^^10^. However, studies focused on the analysis of the influence of other social conditions ^6^ and the identification of high-risk groups^12^ are needed. In this context, it is important to note that FNI in children may compromise caloric and nutrient intake, with possible negative outcomes such as significant growth and development deficiency, compromised health, changes in cognitive development and poor performance at school ^8^^,^^9^. Therefore, this study aimed to analyze the association between the social context (demographic, socioeconomic and social support factors) and moderate-severe food and nutrition insecurity (M-SFNI) in families with children aged 0-59 months enrolled in municipal kindergartens in the state of Paraíba, Brazil.

**KEY MESSAGES**

Motivation for the study. Although current evidence indicates that food and nutritional security is related to socioeconomic conditions, other social context conditions have been little studied.

Main findings. The prevalence of moderate-severe food and nutrition insecurity was 27.2%, mainly in poorer families, beneficiaries of the Bolsa Família Program, those without social support and in dysfunctional families. The lack of interaction, emotional/informational and material support also had a negative influence on food and nutritional security.

Implications. Based on our results, we recommend that the Bolsa Família Program and the social support of families could be improved with social protection mechanisms in order to optimize food and nutritional security.

## MATERIALS AND METHODS

### Design, location and study population

This study is part of the research “NutriESF: Avaliação multifacetada da implantação das ações de alimentação e nutrição na Estratégia Saúde da Família no Nordeste do Brasil” (NutriESF: Multifaceted assessment of the implementation of food and nutrition actions in the Family Health Strategy in Northeast Brazil), which has related articles ^13^. This project is a cross-sectional study on families with children aged 0 to 59 months in their family nucleus, residing in municipalities in the state of Paraíba, Brazil. The project included families in urban areas with children enrolled in municipal public kindergartens, in the period from 2017 to 2018. The project also included a FNI assessment among a set of secondary objectives with representative samples.

The state of Paraíba is located in the west of the northeastern region of Brazil, bordering the states of Rio Grande do Norte to the north, Pernambuco to the south and Ceará to the west. In 2020, the state of Paraíba had an estimated population of 4,039,277 inhabitants ^14^ and a population density of 70.77 inhabitants/km² ^15^. Currently, Paraíba is organized into 16 healthcare regions distributed in three macro-regions, covering its 223 municipalities, in which 1,444 Family Health Strategy (FHS) teams operate, covering 95% of the population ^16^.

We selected ten municipalities (Bayeux, Cabedelo, Cajazeiras, Esperança, Mamanguape, Monteiro, Pombal, Queimadas, São Bento and Sousa) out of 12, with a population between 30,000 and 149,999 inhabitants, which were prioritized for the development of interventions aimed at the prevention of childhood obesity ^17^. Two municipalities were excluded; one because it did not have full FHS coverage, while the other one was used to evaluate the Saúde na Escola Program. The study population included families from the selected municipalities with children aged 0 to 59 months. Since the conditions were related to complications in the child’s health and nutritional status, families with twins, adopted children and mothers under 18 years of age were excluded, in accordance with the primary study protocol.

### Sample

Sample selection was based on probability proportional to the sample size. For each municipality, the number of kindergartens (n = 17) and families (n = 359) participating in the Saúde na Escola Program was determined proportionally according to the number of households with children under five years of age. A sample of 25 families per kindergarten was determined based on the parameters listed above. The institutions were selected first and then the families. In both cases, the selection was by simple random sampling. The records of children from the chosen kindergartens were used for selecting the families.

### Sample size calculation

The sample size was calculated considering a significance level of 95%, a maximum permissible error of 5% and an expected proportion of M-SFNI of 23.7% ^10^, which resulted in the inclusion of at least 276 families of the total number of households with children aged 0 to 59 months in the study municipalities (n=38,140). We added 30% in order to compensate for possible losses and to control for confounding factors, resulting in a required sample of 359 families. A total of 382 families with complete data from the primary study were included.

### Data Collection

Data was collected by interviewers who had previous experience in conducting surveys. The interviewers were undergraduate and graduate students, as well as health professionals. Quality control for the study included: training and standardization of interviewers, construction of an instruction manual, conducting a pilot study in a city from a state that was not included in the research, and supervision of fieldwork. The collected data were organized into electronic spreadsheets and double-entered into a customized database with consistency checks and range restrictions. This database was used for statistical analysis after correction of inconsistent data.

A questionnaire was applied to the mothers, which included questions on the social context of the family and FNS. The social context included the child’s demographic profile (sex and age), socioeconomic status (mother’s work outside the home, family socioeconomic classification and Bolsa Família Program benefit), and social support (mother’s cohabitation with her partner, social support and family functionality).

The socioeconomic classification of the family was based on the criteria of the Brazilian Association of Research Companies, which is used to estimate the buying power of Brazilian families. This classification considers having a bathroom in the household, hiring a maid, the possession of assets, the educational level of the head of household and access to public services. Finally, families were classified into two classes: wealthier (A to C2) or poorer (D-E) ^18^.

The Medical Outcomes Study questionnaire, validated in Brazil, was used for social support. This questionnaire consists of 19 items distributed in five social support dimensions: material (four questions), affective (three questions), emotional (four questions), information (four questions) and social interaction (four questions); the combination of the emotional and information dimensions is recommended. Responses were based on a Likert scale (always, which equals five points; almost always, four points; sometimes, three points; rarely, two points; and never, one point) ^19^. Families were classified as having social support (higher scores) or no social support by k-means cluster analysis.

The Family Apgar questionnaire was used to assess family functionality. Its psychometric properties have been verified in Brazilian families and is recommended in primary health services in Brazil ^20^. This instrument consists of five questions, one for each domain: adaptation, which comprises family resources offered when help is needed; association, which refers to reciprocity in family communication and problem solving; growth, related to the family’s availability for role changes and emotional development; affection, which comprises intimacy and affective interactions in the family context; and resolution, which refers to decision, determination or resolution in a family unit. The questionnaire has three response options (always, which equals two points; sometimes, one point; and never, zero points), with a total score ranging from 0 to 10. Families with scores from 0 to 3 were classified as high family dysfunction, moderate family disfunction was considered as those families with a score from 4 to 6, and functional family as those with a score from 7 to 10 ^20^. For analysis purposes, families were classified as functional or dysfunctional (high and moderate family dysfunction).

FNS was measured with the 14-item version of the Brazilian Food Insecurity Scale (BFIS). The BFIS questions refer to the three months prior to the survey. The number of affirmative responses determines 4 categories: food and nutrition security (0), mild food and nutrition insecurity (1-5), moderate food and nutrition insecurity (6-9), severe food and nutrition insecurity (10-14) ^21^. Households were classified as with or without M-SFNI. In addition, we determined the distribution of households in terms of scale items.

### Variables

M-SFNI was the dependent variable (yes/no). On the other hand, the independent variables were related to child, family and social characteristics. Thus, independent variables included demographic variables of the child such as sex (male, female) and age (0 to 24 months, 25 to 59 months), as well as socioeconomic variables such as maternal work outside the home (yes/no), socioeconomic classification of the family (A-C, D-E) and benefit from the Bolsa Família Program (no/yes). In addition, we included variables related to social support such as cohabitation of the mother with a partner (yes/no), material support (yes/no), affective support (yes/no), emotional/informational support (yes/no), interaction support (yes/no) and family functionality (functional/dysfunctional).

### Statistical Analysis

Population characteristics were described by absolute and relative frequencies. The Pearson’s chi-square test was used to evaluate the differences between the proportions of the M-SFNI and the independent variables. Subsequently, the association of the independent variables and the M-SFNI was determined by using Poisson regression with robust variance to estimate the crude (PR) and adjusted (aPR) prevalence ratios with their respective 95% confidence intervals (95%CI). We included variables with a p-value less than 0.05 (chi-square test) in the crude model. On the other hand, variables with a p-value less than 0.05 in the crude model were included in the adjusted analysis. The variance inflation factor was used to detect multicollinearity, but it was not found (no value was greater than 10). A p-value lower than 0.05 was considered to be statistically significant. We used the statistical program Stata 12.0 (StataCorp LP; College Station, TX, USA).

### Ethical aspects

This research was approved by the Research Ethics Committee of the State University of Paraíba (No. 2.219.620). The mothers of the children who participated in the study signed the free and informed consent form.

## RESULTS

We included 382 families; 17.0% of the children were younger than 25 months. In addition, most families had mothers who did not work outside the home (62.6%), were poorer (68.1%) and benefited by the Bolsa Família Program (78.0%). Regarding social support, affective support was the most frequent (67.3%) and material support was the least frequent (40.0%); while 65.2% of the families were functional. The prevalence of M-SFNI was 27.2%, mostly in families with children under 25 months of age (p=0.025), from poorer condition (p=0.003) and beneficiaries of the Bolsa Família Program (p=0.003). Likewise, deficiencies in material social support (p=0.024), emotional/informational (p=0.020) and interaction (p=0.003), as well as family dysfunctionality (p=0.034) had high proportions of M-SFNI (Table 1).

**Table 1:** Characteristics of families with children aged 0-59 months enrolled in kindergartens according to their moderate-severe food and nutrition insecurity status, Paraíba, Brazil, 2017-2018.

Variables	Total		M-SFNI				p-value ^a^
			No (n=278)		Yes (n=104)		
	n	%	n	%	n	%	
Sex of the child							0.738
Male	171	44.8	123	71.9	48	28.1	
Female	211	55.2	155	73.5	56	26.5	
Age of the child							0.025
25 to 59 months	317	83.0	238	75.1	79	24.9	
0 to 24 months	65	17.0	40	61.5	25	38.5	
Mother works outside the home							0.819
Yes	143	37.4	103	72.0	40	28.0	
No	239	62.6	175	73.2	64	26.8	
Socioeconomic status of the family							0.003
A-C (wealthier classes)	122	31.9	101	82.8	21	17.2	
D-E (poorer classes)	260	68.1	177	68.1	83	31.9	
Bolsa Família Program Benefit							0.003
Yes	298	78.0	206	69.1	92	30.9	
No	84	22.0	72	85.7	12	14.3	
Cohabitation of the mother with a partner							0.109
Yes	252	66.0	190	75.4	62	24.6	
No	130	34.0	88	66.7	42	32.3	
Material support							0.024
Yes	153	40.0	121	79.1	32	20.9	
No	229	60.0	157	68.6	72	31.4	
Emotional support							0.630
Yes	257	67.3	189	73.5	68	26.5	
No	125	32.7	89	71.2	36	28.8	
Emotional/informational support							0.020
Yes	173	45.3	136	78.6	37	21.4	
No	209	54.7	142	67.9	67	32.1	
Interaction support							0.003
Yes	180	47.1	144	80.0	36	20.0	
No	202	52.9	134	66.3	68	33.7	
Family functionality							0.034
Functional family	249	65.2	190	76.3	59	23.7	
Dysfunctional family	133	34.8	88	66.2	45	33.8	

M-SFNI: moderate-severe food and nutrition insecurity.

a p-value calculated using Pearson’s chi-square test.

Table 2 shows the factors associated with M-SFNI. The adjusted analysis showed that families with children younger than 25 months of age (aPR: 1.53; 95%CI: 1.22-1.90), from poorer conditions (aPR: 1.88; 95% CI: 1.60-2.52) and beneficiaries of the Bolsa Família Program (aPR: 2.16; 95% CI: 1.88-2.67) had higher probabilities of presenting M-SFNI. In turn, the lack of material, emotional/informational and interaction support, as well as family dysfunctionality, increased the probability of M-SFNI with aPR values ranging from 1.49 (95%CI: 1.33-1.79) (material support) to 1.73 (95%CI: 1.44-2.01) (interaction support).

**Table 2:** Factors associated with moderate-severe food and nutrition insecurity (M-SFNI) in families with children aged 0-59 months enrolled in kindergartens, Paraíba, Brazil, 2017-2018.

Variables	M-SFNI			
	Crude model		Adjusted model ^a^	
	PR (95%CI)	p-value	aPR (95%CI)	p-value
Age of the child				
25 to 59 months	Ref.		Ref.	
0 to 24 months	1.55 (1.24−1.89)	0.025	1.53 (1.22−1.90)	0.026
Socioeconomic status of the family				
A-C (wealthier)	Ref.		Ref.	
D-E (poorer)	1.85 (1.56−2.54)	0.003	1.88 (1.60−2.52)	0.002
Bolsa Família Program Benefit				
Yes	2.16 (1.88−2.67)	0.003	2.16 (1.88−2.67)	0.003
No	Ref.		Ref.	
Material support				
Yes	Ref.		Ref.	
No	1.50 (1.36−1.78)	0.024	1.49 (1.33−1.79)	0.025
Emotional/Informational Support				
Yes	Ref.		Ref.	
No	1.50 (1.24−1.87)	0.020	1.52 (1.25−1.86)	0.020
Interaction Support				
Yes	Ref.		Ref.	
No	1.69 (1.40−2.06)	0.003	1.73 (1.44−2.01)	0.001
Family Functionality				
Functional family	Ref.		Ref.	
Dysfunctional family	1.43 (1.34−1.90)	0.034	1.43 (1.33−1.89)	0.034

M-SFNI: moderate-severe food and nutrition insecurity; PR: prevalence ratio; aPR: adjusted prevalence ratio; CI: confidence interval; Ref: reference category.

a The adjusted model included all the independent variables with a p-value of less than 0.05 in the crude analysis.

The distribution of households according to the BFIS responses is shown in Table 3. Data for the first four items includes the total study sample (n = 382), while items 5 to 14 include data from families that responded positively to at least one of the items 1 to 4 (n=251). The item regarding the concern with food (running out of food before being able to buy or receive more) had the highest number of positive responses (53.7%) and closest to the FNI classification (65.7%). Item 2 (food ran out before having money to buy more) and item 4 (household members only ate some of the food they had left because money ran out) were answered positively by 26.2% of respondents.

**Table 3:** Frequency of positive responses to questions of the Brazilian Food Insecurity Scale (BFIS) in families with children aged 0-59 months enrolled in kindergartens, Paraíba, Brazil, 2017-2018.

Items	BFIS questions	n^a^	%
1	Household members were concerned that food would run out before they were able to buy or receive more food.	205	53.7
2	Food ran out before family members had money to buy more food.	157	41.1
3	Household members ran out of money for a healthy and varied diet	175	45.8
4	Household members only ate some of the food they had left because money ran out.	184	48.2
5	Any household member aged 18 years or older did not eat a meal because there was no money to buy food.	81	31.9
6	Has any household member aged 18 or older eaten less than he/she thought he/she should because there was no money to buy food.	119	47.0
7	Has any household member 18 years of age or older felt hungry but did not eat because there was no money to buy food?	73	29.1
8	Has any household member 18 years of age or older eaten only one meal a day or gone a whole day without eating because there was no money to buy food?	57	22.7
9	Any household member under 18 years of age stopped eating a healthy and varied diet.	100	39.4
10	Any household member under 18 years of age did not eat enough food because there was no money to buy food.	77	30.7
11	Any household member under 18 years of age reduced the amount of food in meals because there was no money to buy food.	92	36.7
12	Any household member under 18 years of age didn’t have any meal because there was no money to buy food.	57	22.3
13	Any household member under 18 years of age felt hungry, but did not eat, because there was no money to buy food	43	17.1
14	Any household member under 18 years of age has eaten only one meal a day or has gone a whole day without eating because there was no money to buy food	34	13.5

a Items 1 to 4: n = 382, items 5 to 14: n = 251.

## DISCUSSION

Our results show a M-SFNI prevalence of 27.2% in families with children under five in their family nucleus, who attend kindergartens. In addition, we found that families with young children, of lower socioeconomic level and participants of the Bolsa Família Program had a higher probability of M-SFNI. Regarding social support, the probability of M-SFNI was higher for families with limited material, emotional/informational and social interaction support, as well as in dysfunctional families.

The families participating in this study had a socioeconomic configuration marked by social vulnerability, which is an accentuated characteristic of the Brazilian population ^8^^,^^9^. Unfavorable socioeconomic conditions are important determinants of FNI ^6^^-^^10^. The eradication of hunger requires a different view of vulnerable groups as a way to support sustainable development ^1^. In this context, in addition to the social situation, households with children under two years of age expressed another characteristic that requires attention, as these families were more likely to have M-SFNI.

In this study, the prevalence of M-SFNI (27.2%) found in families with children enrolled in kindergartens was slightly higher than that reported by a meta-analysis of samples obtained in school settings between 2004-2013 (23.7%) ^10^ and in the Brazilian population in 2017 (23.0%)^22^. This prevalence rate may be linked to the economic crisis experienced in Brazil from 2014 to 2017 (a period that coincides with the time of data collection in this study), with negative consequences for the social situation of families and access to food, especially among the most vulnerable populations ^22^. This period also coincides with the weakening or dismantling of social protection and food security policies, depriving the poor of the right to food in times of economic decline ^1^^,^^22^. Changes in the FNS situation in Latin American countries and in Brazil during the economic recession (2014 to 2017) have been reported in the literature, even for the most severe degrees of FNI ^23^. Thus, these data reinforce the need for emergency measures to protect and ensure access to food for the most vulnerable families, as well as the constant monitoring of FNS as a way to predict trends and inform decision making in a timely manner.

The socioeconomic conditions that were associated with M-SFNI in our study were the socioeconomic condition of the family and the Bolsa Família Program benefit, which reinforces the extensive literature with emphasis on Latin American countries and particularly Brazil^6^^,^^8^^,^^10^^,^^23^^,^^24^. Similar results have also been reported in other countries such as Ethiopia ^25^. Socioeconomic factors have an impact on the access to food and, therefore, on FNS ^10^. Social indicators may be associated with FNS directly or mediated by income and/or other indicators of the social context ^24^. In turn, the association between M-SFNI and the Bolsa Família Program benefit is based on the social vulnerability of the families receiving this support, delimiting the Program’s inability to improve the social situation and FNI of its public ^10^. Therefore, it is necessary to prioritize social protection programs that really allow increasing buying power and high-quality food to minimize FNI.

Our findings show that mothers who reported lower levels of social support and family functionality belonged to families with a higher probability of having M-SFNI, which agrees with the conceptual model of FNS determinants proposed by Kepple and Segall-Correa ^26^. Other studies have also reported low social support as a factor related to FNI, as is the case of a study conducted in Latin America with 65,146 participants ^23^ and a study involving 107 countries ^27^. In Brazil, studies conducted with nationwide data ^22^ and a population-based study in the metropolitan region of Rio de Janeiro ^28^ also reported similar findings ^22^^,^^28^. FNS can be affected by social support, as it is a strategy for promoting access to adequate quantity and/or quality of food by allowing loans of money or food, support in food production and preparation, help in childcare, building connections to find employment, and emotional support that improves the ability to cope with stressful events ^22^^,^^23^^,^^27^^,^^28^. In addition, social support has been reported to be important for the adherence to health care and services, as well as environmental control and autonomy ^29^. These results highlight social support as a protective factor and the importance of the availability of family and/or friends for FNS, pointing to the need for further research on the subject, given that it is still a recent research topic. Our findings support the importance of providing opportunities for social interaction and companionship as part of the services offered to the community and/or social programs.

Finally, the positive responses to each of the BFIS questions showed that concern with food (53.7%) was one of the items that had one of the highest frequencies of FNI (65.7%), while the frequency of items related to food running out before having money to buy more and members only eating some of the food they had left because money ran out (26.2%) was very close to the prevalence of M-SFNI. These results are similar to those of previous studies in which BFIS questions were considered as a proxy for FNI and its severity ^30^^,^^31^. It is important to note that FNS can be assessed based on isolated BFIS questions, since even a single positive response on the scale classifies the family as FNI. The order of the BFIS questions, from mild to more severe situations, may explain the fact that the first item (concern with lack of food) is the one that best represents FNS ^30^. In turn, the different FNI levels may be represented by aspects related to deprivations in the quantity and quality of food, and not with the concern with the lack of food ^30^^,^^31^. In this sense, a recent study showed that the use of items 2 and 4 of the BFIS as parameters for differentiating FNI levels is useful ^31^, providing credibility to our results regarding the similarity of the prevalence of M-SFNI and the frequency of positive response to these aspects. Thus, we consider that these items should be used to identify early FNI risks and to propose interventions to reverse this situation, including routine health services, social assistance and as a tool for food and nutritional surveillance ^31^^,^^32^.

This study has some limitations. Our results should be generalized with caution, since the sample includes families with specific characteristics. In addition, it is important to note the possibility of residual confounding, as the multifactorial nature of FNI was not fully explored, and potential factors that may act as confounders and contribute to a more complete analyses were not included. The inclusion of data such as children’s food consumption and eating habits, as well as parental interaction with children, not considered in the study, could lead to a broader discussion of the results. From a methodological perspective, it is important to note that the cross-sectional design of this study does not assess causality. However, reverse causality is not so important when dealing with variables, such as those included in this study, that do not change over time and can be collected retrospectively. Additionally, it is pertinent to note that the 2017-2018 indicators presented in this study may not represent the current reality due to the potential consequences of the COVID-19 pandemic in FNS. Regardless, our results are important for future comparisons with studies analyzing the effects of the COVID-19 pandemic on FNS.

Our results showed that M-SFNI was present in 27.2% of families with children aged 0 to 59 months attending kindergartens, being higher in families with children younger than 25 months, of lower socioeconomic level, beneficiaries of the Bolsa Família Program, dysfunctional and without material, emotional/informational and interaction support. Thus, we recommend that the Bolsa Família Program and the social support of families could be improved with social protection mechanisms that can optimize FNS.
