# Supplementary material for: Social context and moderate-severe food and nutritional insecurity in families with children aged 0-59 months, Paraíba, Brazil, 2017-2018
Source: Rev Peru Med Exp Salud Publica. 2023 Mar 29;40(1):7–15. doi: 10.17843/rpmesp.2023.401.12328 (PMC10953632; doi:10.17843/rpmesp.2023.401.12328)
Supplement: Translation

# Translation: Contexto social e Inseguridad Alimentaria y Nutricional Moderada- Grave en familias con niños de 0 a 59 meses, Paraíba, Brasil, 2017- 2018

**Authors:** Dixis Figueroa-Pedraza

**Affiliations:** 1 Universidade Estadual da Paraíba, Campina Grande, Paraíba, Brazil

**Keywords:** Seguridad Alimentaria y Nutricional, Niño, Vulnerabilidad social, Apoyo social, Medio Social, Brasil

## Abstract

**Objetivo.:**

Analizar la asociación entre el contexto social (factores demográficos, socioeconómicos y soporte social) y la inseguridad alimentaria y nutricional moderada-grave de familias con niños de 0 a 59 meses matriculados en jardines infantiles municipales del estado de Paraíba, Brasil.

**Materiales y métodos.:**

Estudio transversal desarrollado en municipios brasileños prioritarios para la prevención de la obesidad infantil. Se utilizó un cuestionario que recogió información sobre el contexto social de la familia (perfil demográfico del niño, situación socioeconómica y soporte social) y la escala brasileña de inseguridad alimentaria. La asociación entre las variables independientes y la inseguridad alimentaria y nutricional moderada-grave se determinó a través de una regresión de Poisson para estimar razones de prevalencia crudas y ajustadas, y sus respectivos intervalos de confianza al 95%.

**Resultados.:**

De un total de 382 familias, el 27,2% presentaba inseguridad alimentaria y nutricional moderada-grave. Además, se encontró que aquellas familias con niños menores de 24 meses, de clases menos prósperas, beneficiadas del *Programa Bolsa Família*, sin apoyo social (material, emocional/informacional y de interacción) y disfuncionales tenían una mayor probabilidad de presentar el desenlace de estudio.

**Conclusiones.:**

Los resultados del presente estudio indicaron que el 27,2% de las familias tenía inseguridad alimentaria y nutricional moderada-grave, siendo mayores en núcleos beneficiarios del *Programa Bolsa Família*, disfuncionales y sin apoyo social. En este sentido, la identificación de estos factores sería de utilidad para optimizar la seguridad alimentaria y nutricional familiar.

## INTRODUCCIÓN

Garantizar la seguridad alimentaria y nutricional (SAN) es una meta global de crucial importancia, tanto para el desarrollo sostenible de las naciones como para la promoción del bienestar nutricional y de la salud de las poblaciones ^1^. Sin embargo, su implementación a nivel local ha sido incipiente, pues en 2020, aún había811 millones de personas en el mundo en situación de hambruna, siendo la mayoría de ellos, niños. Estimaciones realizadas en ese mismo año, sugieren una situación nutricional preocupante en niños menores de cinco años: 149 millones con retraso del crecimiento, 45 millones con insuficiencia ponderal y casi 39 millones con sobrepeso ^2^. Estas cifras indican que será necesario un gran esfuerzo para que el mundo cumpla con las metas relacionadas con la erradicación del hambre y de las diferentes formas de desnutrición, incluyendo a Brasil como signatario de tales propuestas ^1^^,^^2^. En 2018, una quinta parte de las familias brasileñas se encontraba en situación de hambruna ^3^. Además, en 2019, 7,0%, 3,0% y 13,1% de los niños menores de cinco años estaban con talla baja, delgadez y sobrepeso, respectivamente ^4^.

La inseguridad alimentaria y nutricional (IAN) está asociada con la disminución de la ingesta de alimentos, prácticas inadecuadas de alimentación infantil y malas condiciones de salud ^5^. En Brasil, el concepto de SAN manifiesta que la población debe tener un acceso a alimentos garantizado, en cantidad y calidad, sin comprometer otras necesidades esenciales ^6^. De hecho, los factores que determinan los niveles de IAN son múltiples, siendo el bajo poder adquisitivo y la falta de acceso a alimentos nutritivos, los factores más predominantes ^7^^-^^9^.

En una revisión sistemática con meta-análisis de artículos publicados entre 2004 y 2013, se reportó que la prevalencia de IAN entre las poblaciones brasileñas con inequidades sociales fue del 87,2%, evidenciando su determinación social ^10^. Asimismo, para muestras obtenidas en escuelas y jardines infantiles, el estudio mostró que el 61,8% de las familias se encontraba en la misma situación ^10^, a pesar del papel del Programa Nacional de Alimentación Escolar en la garantía de la SAN de los alumnos del sistema público de educación ^11^. Así, los estudios sobre SAN en Brasil han permitido identificar a los factores socioeconómicos como importantes determinantes de esta problemática ^6^^,^^10^. Sin embargo, se requieren estudios enfocados en el análisis de la influencia de otras condiciones del contexto social ^6^^)^ y la identificación de grupos de alto riesgo ^12^. En este contexto, se resalta que la asociación de la IAN al perfil infantil puede comprometer el aporte calórico y de nutrientes, con posibles resultados negativos en los niños como déficits significativos en su crecimiento y desarrollo, comprometer la salud, provocar cambios en el desarrollo cognitivo y disminuir el rendimiento escolar ^8^^,^^9^. Por lo tanto, el objetivo de este estudio fue analizar la asociación entre el contexto social (factores demográficos, socioeconómicos y soporte social) y la inseguridad alimentaria y nutricional moderada-grave (IANM-G) de familias con niños de 0 a 59 meses matriculados en jardines infantiles municipales del estado de Paraíba, Brasil.

**MENSAJES CLAVE**

Motivación para realizar el estudio. A pesar de que, la evidencia actual señala que la seguridad alimentaria y nutricional está relacionada a las condiciones socioeconómicas, se postula que otras condiciones del contexto social han sido poco estudiadas.

Principales hallazgos. La prevalencia de inseguridad alimentaria y nutricional moderada-grave fue 27,2%, siendo que las familias menos prósperas, beneficiarias del Programa Bolsa Família, sin apoyo social y disfuncionales tenían mayor probabilidad de este desenlace. La falta de apoyo material, emocional/informacional y de interacción también influyeron negativamente en la seguridad alimentaria y nutricional.

Implicancias. Estos resultados permitirían recomendar mejoras en el Programa Bolsa Família y en el soporte social de las familias como mecanismos de protección social que pueden optimizar la seguridad alimentaria y nutricional.

## MATERIALES Y MÉTODOS

### Diseño, local y población de estudio

El presente estudio forma parte de los resultados del proyecto de investigación «NutriESF: Avaliação multifacetada da implantação das ações de alimentação e nutrição na Estratégia Saúde da Família no Nordeste do Brasil», sobre el cual, existen publicaciones relacionadas ^13^. Este proyecto es un estudio transversal que investigó a familias con niños de 0 a 59 meses en su núcleo familiar, residentes en municipios del estado de Paraíba, Brasil. El proyecto incluyó a familias de áreas urbanas cuya conformación incluía a niños matriculados en jardines infantiles públicos municipales, en el período de 2017 a 2018. Asimismo, el proyecto incluyó la evaluación de la IAN entre un conjunto de objetivos secundarios con muestras representativas. 

El estado de Paraíba está localizado al oeste de la región nordeste de Brasil, limitándose con los estados de Rio Grande do Norte al norte, Pernambuco al sur y Ceará al oeste. En 2020, el estado de Paraíba tenía una población estimada de 4 039 277 habitantes ^14^ y una densidad demográfica de 70,77 hab/km² ^15^. La configuración actual de la regionalización de la salud en Paraíba organiza el estado en 16 regiones de salud distribuidas en tres macrorregiones, que abarcan sus 223 municipios, en las que actúan 1,444 equipos de la Estrategia de Salud de la Familia (ESF), con una cobertura del 95% de la población ^16^.

Para el propósito del estudio, se seleccionaron diez municipios (Bayeux, Cabedelo, Cajazeiras, Esperança, Mamanguape, Monteiro, Pombal, Queimadas, São Bento y Sousa) de un total de 12, con una población entre 30,000 y 149,999 habitantes, que fueron prioritarios para el desarrollo de acciones dirigidas a la prevención de la obesidad infantil ^17^. Dos de los municipios fueron excluidos. Uno de ellos debido a que era el único que no tenía cobertura total de la ESF, mientras que el otro se utilizó para evaluar el *Programa Saúde na Escola*. Se consideró como población de estudio a las familias de los municipios seleccionados con niños de 0 a 59 meses. Por tratarse de afecciones relacionadas a complicaciones en el estado de salud y nutrición del niño, se excluyó del estudio a las familias con niños gemelos, adoptados y madres menores de 18 años, conforme al protocolo del estudio primario.

### Muestra

La selección de la muestra se basó en la probabilidad proporcional al tamaño de la muestra. Para cada municipio, el número de jardines infantiles (n = 17) y de familias (n = 359) participantes del Programa Saúde na Escola se estableció proporcionalmente en función de la cantidad de domicilios con menores de cinco años. Además, basado en los parámetros anteriores, se estableció una muestra por jardín infantil de 25 familias. En primer lugar, se seleccionaron a las instituciones y posteriormente a las familias. En ambos casos, la selección fue por muestreo aleatorio simple. Con los jardines infantiles escogidos, para la selección de las familias se utilizaron los registros de los niños disponibles en esas instituciones.

### Cálculo del tamaño de muestra

El cálculo del tamaño de muestra se realizó considerando un nivel de significancia de 95%, error máximo permisible del 5% y una proporción esperada de IANM-G del 23,7% ^10^, lo que resultó en la necesidad de incluir al menos a 276 familias del total de domicilios con niños de 0 a 59 meses en los municipios de estudio (n=38,140). A esta cifra se sumó el 30% para compensar posibles pérdidas y controlar factores de confusión, siendo la muestra requerida de 359 familias. Para el análisis, se tuvo la disponibilidad de un total de 382 familias con datos completos del estudio primario.

### Recolección de datos

La información de los participantes fue recogida por entrevistadores que tenían experiencia previa en la realización de encuestas. Los encuestadores estaban constituidos por estudiantes de pregrado y posgrado, así como profesionales de la salud. El control de calidad del estudio incluyó: capacitación y estandarización de entrevistadores, construcción de un manual de instrucción, realización de un estudio piloto en una ciudad del estado que no participó de la investigación y supervisión del trabajo de campo. Los datos recopilados se organizaron en hojas de cálculo electrónicas y se digitaron en entrada doble, en una base de datos personalizada, con controles de coherencia y restricciones de rango. La base de datos producida después de la corrección de los datos con inconsistencias se utilizó para el análisis estadístico.

Para la obtención de los datos, se aplicó un cuestionario a las madres que incluyó preguntas sobre el contexto social de la familia y la SAN. El contexto social comprendió el perfil demográfico del niño (sexo y edad), la situación socioeconómica (trabajo de la madre fuera del hogar, clasificación socioeconómica de la familia y beneficio del *Programa Bolsa Família*), y el soporte social (convivencia de la madre con su compañero, apoyo social y funcionalidad familiar).

La clasificación socioeconómica de la familia se basó en el criterio de la Asociación Brasileña de Empresas de Investigación, que se utiliza para estimar el poder adquisitivo de las familias brasileñas. Esta clasificación considera la existencia de un baño en el hogar, la contratación de una empleada doméstica, la posesión de bienes, el nivel educativo del jefe de familia y el acceso a los servicios públicos. Finalmente, las familias se clasificaron en dos clases: clases más prósperas (de A a C2) o menos prósperas (D-E) ^18^.

Para el apoyo social se utilizó el cuestionario, validado en Brasil, *Medical Outcomes Study*. Este cuestionario consta de 19 ítems distribuidos en cinco dimensiones de apoyo social: material (cuatro preguntas), afectivo (tres preguntas), emocional (cuatro preguntas), información (cuatro preguntas) e interacción social (cuatro preguntas); recomendándose la agregación de las dimensiones emocional e información. Las respuestas a las preguntas se basan en una escala de Likert (siempre, que equivale a cinco puntos; casi siempre, a cuatro puntos; a veces, a tres puntos; rara vez, a dos puntos; y nunca igual a un punto) ^19^. Las familias fueron clasificadas con apoyo social (mayores puntuaciones) o sin apoyo social por medio de un análisis de clúster (k-means).

Para evaluar la funcionalidad familiar se utilizó el cuestionario Apgar de la Familia, cuyas propiedades psicométricas han sido verificadas en familias brasileñas y su uso se recomienda en los servicios primarios de salud en Brasil ^20^. Este instrumento consta de cinco preguntas, una para cada dominio de evaluación: adaptación, que comprende los recursos familiares ofrecidos cuando se necesita ayuda; asociación, que se refiere a la reciprocidad en la comunicación familiar y la resolución de problemas; crecimiento, relacionado con la disponibilidad de la familia para cambios de roles y desarrollo emocional; afecto, que comprende la intimidad y las interacciones afectivas en el contexto familiar, y resolución, que se refiere a la decisión, determinación o resolución en una unidad familiar. El cuestionario tiene tres opciones de respuesta (siempre, que equivale a dos puntos; a veces, un punto, y nunca que equivale a cero puntos), con una puntuación total que va de 0 a 10. Las familias con puntuaciones de 0 a 3 se clasificaron como alta disfunción familiar; de 4 a 6, disfunción familiar moderada, y de 7 a 10, familia funcional ^20^. Para fines del análisis, las familias fueron clasificadas en funcionales o disfuncionales (disfunción familiar alta y moderada).

La SAN se midió con la Escala Brasileña de Inseguridad Alimentaria (EBIA), utilizando la versión de 14 ítems. Las preguntas de la EBIA se refieren a los tres meses anteriores a la encuesta, y el número de respuestas afirmativas determina la clasificación en: seguridad alimentaria y nutricional (0), inseguridad alimentaria y nutricional leve (1-5), inseguridad alimentaria y nutricional moderada (6-9), inseguridad alimentaria y nutricional grave (10-14) ^21^. Para el análisis, los hogares fueron clasificados con o sin IANM-G. Además, se determinó la distribución de las familias en cuanto a los ítems de la escala.

### Variables

La variable dependiente fue la IANM-G (sí, no). Por otro lado, las variables independientes se relacionaron con las características del niño, sus familias y sociales. Así, como variables independientes se incluyeron a variables demográficas del niño como sexo (masculino, femenino) y edad (0 a 24 meses, 25 a 59 meses), así como de la situación socioeconómica como trabajo materno fuera del hogar (sí, no), clasificación socioeconómica de la familia (A-C, D-E) y beneficio del *Programa Bolsa Família* (no, sí). Además, se incluyeron variables relacionadas con el soporte social como convivencia de la madre con su compañero (sí, no), apoyo material (sí, no), apoyo afectivo (sí, no), apoyo emocional/informacional (sí, no), apoyo de interacción (sí, no) y funcionalidad familiar (funcional, disfuncional).

### Análisis Estadístico

Las características de la población se describieron mediante frecuencias absolutas y relativas. Además, las diferencias de las proporciones entre la IANM-G y las variables independientes fue evaluado mediante la prueba de chi-cuadrado de Pearson. Posteriormente, se determinó la asociación de las variables independientes con la IANM-G a través de una regresión de Poisson con varianza robusta para estimar las razones de prevalencia crudas (RP) y ajustadas (RPa) con sus respectivos intervalos de confianza al 95% (IC 95%). En el modelo crudo, se incluyeron a las variables que tuvieron un valor de p menor a 0.05 en la prueba chi-cuadrado, mientras que en el análisis ajustado se incluyeron a las variables con un valor de p menor a 0.05 en el análisis crudo. La ocurrencia de multicolinealidad fue verificada por el factor de inflación de la variancia, antes de la regresión, no siendo detectada (ningún valor fue superior a 10). Se consideró un valor de p<0.05 para delimitar significancia estadística en todos los análisis. Los análisis se realizaron con el programa estadístico Stata 12.0 (StataCorp LP; College Station, TX, USA).

### Aspectos éticos

El proyecto de investigación fue aprobado por el Comité de Ética en Investigación de la Universidad Estadual de Paraíba (No. 2.219.620). Las madres de los niños que participaron en el estudio firmaron el formato de consentimiento libre e informado.

## RESULTADOS

Un total de 382 familias fueron incluidas en el estudio, de las cuales, el 17,0% de los niños eran menores de 25 meses. Además, hubo un predominio de familias con madres que no trabajaban fuera del hogar (62,6%), clasificadas en las clases menos prósperas (68,1%) y beneficiadas por el *Programa Bolsa Família* (78,0%). En relación con el apoyo social, se destacó una mayor frecuencia para el dominio afectivo (67,3%) y menor para el material (40,0%); mientras que el 65,2% de las familias eran funcionales. La prevalencia de IANM-G fue del 27,2%, con mayores proporciones en familias con niños menores de 25 meses (p=0,025), de clases menos prósperas (p=0,003) y beneficiadas del *Programa Bolsa Família* (p=0,003). Para las variables relacionadas al soporte social, las deficiencias en apoyo social material (p=0,024), emocional/informacional (p=0,020) y de interacción (p=0,003), así como la disfuncionalidad familiar (p=0,034), representaron condiciones con mayores proporciones de IANM-G (Tabla 1).

**Tabla 1:** Características de familias con niños de 0 a 59 meses matriculados en jardines infantiles según su situación de inseguridad alimentaria y nutricional moderada-grave, Paraíba, Brasil, 2017-2018.

Variables	Total		IANM-G				Valor de p ^a^
			No (n=278)		Si (n=104)		
	n	%	n	%	n	%	
Sexo del niño							0,738
Masculino	171	44,8	123	71,9	48	28,1	
Femenino	211	55,2	155	73,5	56	26,5	
Edad del niño							0,025
25 a 59 meses	317	83,0	238	75,1	79	24,9	
0 a 24 meses	65	17,0	40	61,5	25	38,5	
Trabajo materno fuera de casa							0,819
Sí	143	37,4	103	72,0	40	28,0	
No	239	62,6	175	73,2	64	26,8	
Clasificación socioeconómica de la familia							0,003
A-C (clases más prósperas)	122	31,9	101	82,8	21	17,2	
D-E (clases menos prósperas)	260	68,1	177	68,1	83	31,9	
Beneficio del Programa Bolsa Família							0,003
Sí	298	78,0	206	69,1	92	30,9	
No	84	22,0	72	85,7	12	14,3	
Convivencia de la madre con el compañero							0,109
Sí	252	66,0	190	75,4	62	24,6	
No	130	34,0	88	66,7	42	32,3	
Apoyo material							0,024
Sí	153	40,0	121	79,1	32	20,9	
No	229	60,0	157	68,6	72	31,4	
Apoyo afectivo							0,630
Sí	257	67,3	189	73,5	68	26,5	
No	125	32,7	89	71,2	36	28,8	
Apoyo emocional/informacional							0,020
Sí	173	45,3	136	78,6	37	21,4	
No	209	54,7	142	67,9	67	32,1	
Apoyo de interacción							0,003
Sí	180	47,1	144	80,0	36	20,0	
No	202	52,9	134	66,3	68	33,7	
Funcionalidad familiar							0,034
Familia funcional	249	65,2	190	76,3	59	23,7	
Familia disfuncional	133	34,8	88	66,2	45	33,8	

IANM-G: inseguridad alimentaria y nutricional moderada-grave.

a Valor de p calculado mediante la prueba chi-cuadrado de Pearson.

La Tabla 2 muestra los factores asociados con la IANM-G. Los resultados referidos al análisis ajustado muestran que las familias con niños menores de 25 meses (RPa: 1,53; IC 95%: 1,22-1,90), de clases menos prósperas (RPa: 1,88; IC 95%: 1,60-2,52) y beneficiarias del Programa Bolsa Família (RPa: 2,16; IC 95%: 1,88-2,67) presentaron probabilidades mayores de IANM-G. A su vez, la falta de apoyo material, emocional/informacional y de interacción, así como la disfuncionalidad familiar, representaron condiciones que aumentarían la probabilidad de IANM-G con valores de RPa que oscilan entre 1,49 (IC 95%: 1,33-1,79) (apoyo material) y 1,73 (IC 95%: 1,44-2,01) (apoyo de interacción).

**Tabla 2:** Factores asociados con la inseguridad alimentaria y nutricional moderada-grave (IANM-G) en familias con niños de 0 a 59 meses matriculados en jardines infantiles, Paraíba, Brasil, 2017-2018.

Variables	IANM-G			
	Modelo Crudo		Modelo Ajustado ^a^	
	RP (IC 95%)	Valor de p	RPa (IC 95%)	Valor de p
Edad del niño				
25 a 59 meses	Ref.		Ref.	
0 a 24 meses	1,55 (1,24−1,89)	0,025	1,53 (1,22−1,90)	0,026
Clasificación socioeconómica de la familia				
A-C (clases más prósperas)	Ref.		Ref.	
D-E (clases menos prósperas)	1,85 (1,56−2,54)	0,003	1,88 (1,60−2,52)	0,002
Beneficio del Programa Bolsa Família				
Sí	2,16 (1,88−2,67)	0,003	2,16 (1,88−2,67)	0,003
No	Ref.		Ref.	
Apoyo Material				
Sí	Ref.		Ref.	
No	1,50 (1,36−1,78)	0,024	1,49 (1,33−1,79)	0,025
Apoyo Emocional/Informacional				
Sí	Ref.		Ref.	
No	1,50 (1,24−1,87)	0,020	1,52 (1,25−1,86)	0,020
Apoyo de Interacción				
Sí	Ref.		Ref.	
No	1,69 (1,40−2,06)	0,003	1,73 (1,44−2,01)	0,001
Funcionalidad Familiar				
Familia funcional	Ref.		Ref.	
Familia disfuncional	1,43 (1,34−1,90)	0,034	1,43 (1,33−1,89)	0,034

IANM-G: inseguridad alimentaria y nutricional moderada-grave, RP: razón de prevalencia, RPa: razón de prevalencia ajustada, IC: intervalo de confianza, Ref.: categoría de referencia.

a El modelo ajustado incluyo a todas las variables independientes que obtuvieron un valor de p menor a 0.05 en el análisis crudo.

La distribución de las familias en cuanto a las respuestas de los ítems de la EBIA se observa en la Tabla 3. Para los primeros cuatros ítems, los valores se refieren a la muestra total de estudio (n = 382), mientras que para los ítems 5 a 14 se consideraron las familias que respondieron positivamente a al menos uno de los ítems 1 a 4 (n = 251). La cuestión referente a la preocupación con la alimentación (acabar los alimentos antes de poder comprar o recibir más) fue la que presentó mayor cantidad de respuestas positivas (53,7%) y más próxima de la clasificación de IAN (65,7%). El ítem 2 (los alimentos acabaron antes de tener dinero para comprar más) y el ítem 4 (los miembros del hogar solo comieron algunos alimentos que les quedaba porque el dinero acabó) fueron respondidos positivamente por 26,2% de los entrevistados.

**Tabla 3:** Frecuencia de respuestas positivas a las preguntas de la Escala Brasileña de Inseguridad Alimentaria (EBIA) en familias con niños de 0 a 59 meses matriculados en jardines infantiles, Paraíba, Brasil, 2017-2018.

Ítems	Cuestiones de la EBIA	n^a^	%
1	A los miembros del hogar les preocupaba que los alimentos se acabaran antes de que pudieran comprar o recibir más alimentos	205	53,7
2	Los alimentos se agotaron antes de que los miembros de la familia tuvieran dinero para comprar más comida	157	41,1
3	Los miembros del hogar se quedaron sin dinero para tener una alimentación saludable y variada	175	45,8
4	Los miembros del hogar solo comieron algunos alimentos que les quedaba porque se acabó el dinero	184	48,2
5	Algún miembro del hogar de 18 años o más no realizó alguna alimentación porque no había dinero para comprar alimentos	81	31,9
6	Algún miembro del hogar de 18 años o más ha comido menos de lo que pensaba que debería porque no había dinero para comprar alimentos	119	47,0
7	Algún miembro del hogar de 18 años o más ha sentido hambre pero no comió porque no había dinero para comprar alimentos	73	29,1
8	Algún miembro del hogar de 18 años o más ha comido una sola alimentación al día o ha pasado un día entero sin comer porque no había dinero para comprar alimentos	57	22,7
9	Algún miembro del hogar menor de 18 años dejó de tener una alimentación saludable y variada	100	39,4
10	Algún miembro del hogar menor de 18 años no comió lo suficiente porque no había dinero para comprar alimentos	77	30,7
11	Algún miembro del hogar menor de 18 años redujo la cantidad de alimentos en las alimentaciones porque no había dinero para comprar alimentos	92	36,7
12	Algún miembro del hogar menor de 18 años dejó de hacer alguna alimentación porque no había dinero para comprar alimentos	57	22,3
13	Algún miembro del hogar menor de 18 años sintió hambre, pero no comió, porque no había dinero para comprar alimentos	43	17,1
14	Algún miembro del hogar menor de 18 años ha comido solo una alimentación al día o ha pasado un día entero sin comer porque no había dinero para comprar alimentos	34	13,5

a Ítems 1 a 4: n = 382, ítems 5 a 14: n = 251.

## DISCUSIÓN

Los resultados del estudio muestran una prevalencia de IANM-G del 27,2% en familias con niños menores de cinco que frecuentan jardines infantiles dentro el núcleo familiar. Además, se encontró que las familias con niños pequeños, de nivel socioeconómico más bajo y participantes del *Programa Bolsa Família* tuvieron una mayor probabilidad de IANM-G. Respecto a las variables del soporte social, la probabilidad de IANM-G fue mayor para las familias con apoyo material, emocional/informacional y de interacción social limitado, así como en las disfuncionales.

Las familias participantes de este estudio tenían una configuración socioeconómica marcada por la vulnerabilidad social, que es una característica acentuada de la población brasileña ^8^^,^^9^. Las condiciones socioeconómicas desfavorables son determinantes importantes de la IAN ^6^^-^^10^. La erradicación del hambre presupone una visión diferenciada de los grupos en situación de vulnerabilidad como forma de apoyar el desarrollo sostenible ^1^. En el contexto estudiado, además de la situación social, los núcleos familiares con niños menores de dos años expresaron otra característica sobre la cual se debe centrar la atención, ya que estas familias tenían mayor probabilidad de presentar IANM-G.

En este estudio, la prevalencia de IANM-G (27,2%) encontrada en familias con niños matriculados en jardines infantiles fue ligeramente superior a la reportada a través de un meta-análisis para muestras obtenidas en ambiente escolar entre 2004-2013 (23,7%) ^10^ y en la población brasileña en 2017 (23,0%) ^22^. Esta prevalencia puede estar vinculada a la crisis económica vivenciada en Brasil de 2014 hasta 2017 (año que coincide con la época de la recolección de datos de este estudio), con consecuencias negativas para la situación social de las familias y el acceso a los alimentos, especialmente entre los más vulnerables ^22^. Este período también coincide con el debilitamiento o desmantelamiento de las políticas de protección social y seguridad alimentaria, privando a los pobres del derecho a la alimentación en tiempos de declive económico ^1^^,^^22^. Los cambios en la situación de la SAN en los países de América Latina y en Brasil durante la recesión económica (2014 a 2017) han sido mostrados en la literatura, incluso para los grados más severos de IAN ^23^. Así, estos datos refuerzan la necesidad de medidas de emergencia para proteger y garantizar el acceso a la alimentación de las familias más vulnerables, así como el monitoreo constante de la IAN como forma de predecir tendencias y fundamentar la toma de decisiones de manera oportuna.

Las condiciones socioeconómicas que se asociaron a la IANM-G en el presente estudio fueron la clasificación socioeconómica de la familia y el beneficio del *Programa Bolsa Família*, lo que refuerza la extensa literatura con ese énfasis en los países de América Latina y en Brasil ^6^^,^^8^^,^^10^^,^^23^^,^^24^. También se han informado resultados similares en otros países como Etiopía ^25^. Los factores socioeconómicos inciden en el nivel de acceso a los alimentos y, por lo tanto, en la SAN ^10^. Los indicadores sociales pueden estar asociados a la IAN de forma directa o mediados por el ingreso y/u otros indicadores del contexto social ^24^. A su vez, la asociación entre la IANM-G y el beneficio del *Programa Bolsa Família* se justifica con base en la vulnerabilidad social de las familias que reciben ese apoyo, delimitando la incapacidad del Programa para mejorar la situación social y la IAN de su público ^10^. Por lo tanto, es necesario priorizar programas de protección social que realmente permitan aumentar el poder adquisitivo y de alimentos de calidad para minimizar la situación de la IAN.

Con respecto al soporte social, los hallazgos de esta investigación revelaron que las madres que reportaron peores niveles de apoyo social y funcionalidad familiar representaban familias con mayor probabilidad de padecer IANM-G, lo que está en concordancia con el modelo conceptual de determinantes de la SAN propuesto por Kepple y Segall-Correa ^26^. Resultados de otros estudios también han mostrado el bajo apoyo social como un factor relacionado con la IAN, como es el caso de una investigación realizada en América Latina que incluyó a 65,146 encuestados ^23^ y un estudio que involucró a 107 países ^27^. En Brasil, análisis realizados con datos a nivel nacional ^22^ y un estudio de base poblacional desarrollado en la región metropolitana de Río de Janeiro ^28^ también destacaron hallazgos similares ^22^^,^^28^. La SAN puede afectarse por el apoyo social, ya que este último factor constituye una estrategia de promoción del acceso a los alimentos en cantidad y/o calidad al posibilitar el préstamo de dinero o alimentos, apoyo en la producción y preparación de alimentos, ayuda en el cuidado de los niños, la formación de conexiones para encontrar empleo y apoyo emocional que mejora la capacidad de afrontar eventos estresantes ^22^^,^^23^^,^^27^^,^^28^. Además, el apoyo social también ha sido destacado por su importancia en la adhesión a los cuidados y servicios de salud, así como en el control del ambiente y en la autonomía ^29^. Estos resultados destacan el apoyo social como factor de protección y la importancia de la disponibilidad de familiares y/o amigos para la SAN, apuntando para la necesidad de profundizar la temática, dado que aún es un asunto de investigación reciente. Nuestros hallazgos respaldan la importancia de proporcionar oportunidades para la interacción social y el compañerismo como parte de los servicios ofrecidos a la comunidad y/o de los programas sociales.

Finalmente, el análisis de las respuestas positivas a cada una de las preguntas de la EBIA apuntó que la preocupación con la alimentación (53,7%) fue uno de los ítems que presentó una de las frecuencias más altas de la IAN (65,7%), mientras que los ítems relacionados a que los alimentos acabaron antes de tener dinero para comprar más y sus miembros solo comieron algunos alimentos que les quedaba porque el dinero acabó (26,2%) presentaron una frecuencia muy próxima a la prevalencia de IANM-G. Esos resultados convergen con los de otros estudios en los cuales preguntas de la EBIA han sido consideradas como *proxy* de la IAN y su gravedad ^30^^,^^31^. Para la primera situación se destaca que la SAN puede ser evaluada basándose en preguntas aisladas de la EBIA, pues una única respuesta positiva de la escala ya clasifica la familia en IAN. El orden de las preguntas de la EBIA, de situaciones más leves a más severas, puede explicar el hecho de ser el primer ítem (preocupación con la falta de alimentos) el que represente de mejor forma a la IAN ^30^. A su vez, los diferentes niveles de IAN pueden ser representados por aspectos relacionados con privaciones en la cantidad y calidad de los alimentos, y no con la preocupación por la falta de los mismos ^30^^,^^31^. En ese sentido, un estudio reciente mostró la adecuación del uso de los ítems 2 y 4 de la EBIA como parámetros para la discriminación de los niveles de IAN ^31^, transmitiendo plausibilidad a los resultados del presente trabajo con relación a la proximidad de la prevalencia de IANM-G y la frecuencia de respuesta positiva a esos aspectos. Así, se recomienda que esos ítems sean utilizados para identificar precozmente riesgos de IAN y proponer intervenciones que posibiliten revertir ese cuadro, incluso en la rutina de los servicios de salud, en la asistencia social y como instrumento de vigilancia alimentar y nutricional ^31^^,^^32^.

Este estudio tiene limitaciones. La generalización de sus resultados debe realizarse con cautela, ya que la muestra investigada representa familias con criterios específicos. Además, se debe considerar la posibilidad de confusión residual, ya que la naturaleza multifactorial de la IAN no fue completamente explorada, al no incluirse factores potenciales que pueden actuar como confusores y contribuir con análisis más completos. La inclusión de datos como el consumo y los hábitos alimentarios de los niños, así como la interacción de los padres con los hijos, no considerados en el estudio, podrían permitir la discusión de los resultados desde una perspectiva más amplia. Desde una perspectiva metodológica, se sabe que el diseño transversal adoptado no permite evaluar la causalidad. Sin embargo, la causalidad inversa no es tan importante cuando se trata de variables, como las que se exploraron en este trabajo, que no cambian en el tiempo y se pueden recolectar retrospectivamente. Adicionalmente, es pertinente señalar que los indicadores de 2017-2018 mostrados en este estudio pueden no representar la realidad actual debido a las potenciales consecuencias de la pandemia de COVID-19 en la SAN. A pesar de eso, los resultados encontrados también son importantes para realizar futuras comparaciones con estudios que analicen los efectos de la pandemia de COVID-19 en la SAN.

Los resultados del presente estudio indicaron que la IANM-G estuvo presente en el 27,2% de familias con niños de 0 a 59 meses que asisten a jardines infantiles, siendo mayores en núcleos con niños menores de 25 meses, de menor nivel socioeconómico, beneficiarios del *Programa Bolsa Família*, disfuncionales y sin apoyo material, emocional/informacional y de interacción. Así, se recomienda mejorías en el *Programa Bolsa Família* y en el soporte social de las familias como mecanismos de protección social que pueden optimizar su SAN.
